# Management of Enterocutaneous Fistula: A Review

**DOI:** 10.31729/jnma.5780

**Published:** 2022-01-31

**Authors:** Pradeep Ghimire

**Affiliations:** 1 Department of Surgery, Manipal College of Medical Sciences, Fulbari, Pokhara, Nepal

**Keywords:** *drainage*, *enteral nutrition*, *intestinal fistula*, *malnutrition*, *sepsis*

## Abstract

Enterocutaneous fistula is any communication between bowel and skin or atmosphere outside the body. It can be classified by various means by etiology, organ of origin, etc. Enterocutaneous fistula can occur after any gastrointestinal surgery where there is some trauma during surgery or other associated causes such as malignancy, inflammatory bowel disease, foreign body, etc. Enterocutaneous fistula needs a multidisciplinary approach as its management is a very tedious and complex process. Sepsis, malnutrition, and dyselectrolytemia are three key factors during the management of enterocutaneous fistula, so these should be properly addressed for better and efficient outcomes. There is excess fistula effluent which should be replaced adequately in high output fistula. The nutrition of the patient plays a vital role in the success of enterocutaneous fistula management so if the patient can tolerate oral or enteral feeding should be commenced as soon as possible otherwise parenteral nutrition should be advised. Wound care should be done aggressively, proper skincare, timely drainage of any localised abscesses should be done. Patients should be properly resuscitated and stabilised before any definitive investigations and management. Surgical therapy can be staged and should not be rushed which results in failure of this complex disease process.

## INTRODUCTION

An enterocutaneous fistula (ECF) is an abnormal connection between the intra-abdominal gastrointestinal (GI) tract and skin/wound.^1^ Enterocutaneous fistula is associated with substantial morbidity and mortality and significant patient distress. Sepsis and malnutrition are the chief cause of death. The initial focus for the treatment should be treating the fluid and electrolyte disturbances, aggressive treatment of sepsis, control of fistula output, and attention to skincare and psychological support.^2^ The management of ECF is a complex and tedious process with a multidisciplinary approach. For most postoperative fistulas, further surgery is planned only if the fistula persists after conservative measures. This article reviewed the justification and evidence behind the current management approach for enterocutaneous fistula. The goal is the closure of the fistula with minimal morbidity and mortality.

## CLASSIFICATION

ECF is often classified according to total output, etiology, and source. A high-output ECF has output >500mL/24 hours, a moderate output fistula between 200 to 500mL/24 hours, and a low output <200mL/24 hours.^1^ The common causes of iatrogenic ECF are trauma; operations for malignant disease with extensive adhesiolysis or associated inflammatory bowel disease (IBD); and trauma.^2^ The majority of ECFs are iatrogenic (75-85%), and 15 to 25% occur spontaneously. Of postoperative small bowel fistulae, half are from an anastomotic leak, and the other half occur due to inadvertent injury to the small bowel during dissection.^1^ The common causes of spontaneous fistulae occur due to IBD (most common), malignancy, appendicitis, diverticulitis, radiation, tuberculosis/ actinomycosis, and ischemia.^3,4^

An ECF can occur as a complication of a GI tract surgery of any type. More than 75% of all ECFs arise as a postoperative complication, whereas about 15-25% result from abdominal trauma or occur spontaneously about cancer, irradiation, inflammatory bowel disease (IBD), or ischemic or infective conditions. The etiology of ECFs can thus be characterized as postoperative, traumatic, or spontaneous.^5^

Anatomically ECF can be divided into internal and external fistulas and this helps in identifying the organs involved and providing characteristics of the fistula tract. Internal fistulas have communications between two hollow viscera which if symptomatic should be treated by resection and re-anastomosis.^3^ The external fistulas connect hollow viscera to the skin. External fistulas that have favourable outcomes include oesophageal, duodenal stump, pancreaticobiliary, and jejunal fistulas with small enteric defects (<1cm) and long tracts (>2cm). Gastric, lateral duodenal, ligament of Treitz, and ileal fistulas are less favourable to close spontaneously, as are fistulas are related to complete disruption of intestinal continuity, adjacent abscess, diseased bowel, foreign bodies, or distal obstruction.

According to the organ of origin ECF can be accustomed into type I (abdominal, oesophageal, gastroduodenal), type II (small bowel), type III (large bowel), and type IV (enter atmospheric, regardless of origin).^5^ Edmunds et al. acknowledged the classic triad of complications of enterocutaneous fistula as sepsis, malnutrition, and electrolyte and fluid abnormalities. The presence of bowel contents outside the lumen may lead to a localized abscess, infection over soft tissues, generalized peritonitis, or sepsis, reliant on whether the bowel leak communicates with the peritoneal cavity or soft tissues. Initial control of fistula output, drainage of confined collections, and suitable antibiotic therapy are keys in the early management of these patients. Recent studies advocate that sepsis, associated with malnutrition, is the foremost cause of death in patients with enterocutaneous fistulas.^6,7^

To catch up for the loss of continuity with the distal part of the bowel, the GI tract undergoes three phases of bowel adaptation. ^7,8^

1. Hypersecretory phase: This phase occurs up to three days after stoma formation and might last for 1 to 2 months. It is characterized by large volume losses that might be bilious.
2. Adaptation phase: This phase starts three to five days after stoma formation and may last for up to 12 months. It is characterized by a decrease in output. The speed of adaptation relies on the age of the patient, the severity of the underlying disease, and the site of small bowel resection.
3. Stabilization phase: It is characterized by a further decrease in fluid loss and eventual stabilization of stoma output. This phase may take up to 24 months.

## MANAGEMENT

Particularly, for the patient with acute intestinal failure in whom there is an enterocutaneous fistula the Maastricht group has proposed the SOWATS regimen for enterocutaneous fistula or temporary enterostomy.^9^

S = Sepsis control
O = Optimisation of nutritional status
W = Wound care
A = Anatomy of the bowel and the fistula
T = Timing of surgery
S = Surgical planning

Very similar guidance has come from the Salford unit in the UK, with the acronym SNAP: for Sepsis, Nutrition, Anatomy, Plan.^9,10^

Management of a gastrointestinal fistula could be a difficult and sophisticated process. However, a scientific approach may result in treatment that is effective and potentially rewarding. In general, management could also be compartmentalized into five stages: stabilization, investigation, decision, definitive therapy, and healing.^11,12^

The SOWATS treatment guideline consists of the subsequent components: Sepsis, Optimization of nutritional state, Wound care, Anatomy (of the fistula), Timing of surgery, and Surgical strategy.^13^

Due to fluid losses from the fistula and third space fluid loss there is profound hypovolemia, so restoration of the intravascular fluid should be the primary priority.^11^ Treatment of enterocutaneous fistulae could be a challenge and within the majority of patients, complex multiple therapies are needed.^14^ The effect of sepsis on survival is multifactorial. Active infection results in impairment of nutrient transport, bowel motility, the proliferation of enterocytes, and mucosal barrier function.^15^

## RESUSCITATION

Resuscitation of a patient with a newly diagnosed ECF follows many of the identical principles because the resuscitation of septic patients and tenets of the Surviving Sepsis Campaign should function as a framework.^16,17^ Initial care should focus on aggressive fluid resuscitation, rapid assessment and correction of electrolyte imbalances, and normalization of lactic acidosis. Patients with ECF are commonly hyponatremic, hyperkalemic, and acidotic because of ongoing GI losses. Patients with high output fistulas should have fluid, electrolyte, and bicarbonate losses replaced intravenously to avoid dehydration and profound metabolic instability during the initial stabilization period. Careful monitoring of urine output and targeted replacement of fistula effluent every four to eight hours will prevent ongoing dehydration.^17^ Significant supplementation of electrolytes like sodium, potassium, and magnesium is required. Magnesium deficiency may cause nausea, apathy, and neuromuscular hyperexcitability.^18^ Normal saline with potassium chloride is used for replacing fistula losses from the small intestine in high fistula type.

Malnutrition remains a serious clinical problem of ECF, so nutrition plays a significant role in ECF management and helps reduce the morbidity and mortality of patients, mainly in high output fistula or with associated septic complications.^10,19^

It is advised to start nutritional support as soon as the patient is stabilized. Full caloric and nitrogen replacement are often provided within some days. Blood transfusion is also required if there is significant anaemia which may be due to the premorbid condition, postoperative, chronic blood loss, malnutrition, or the bone-marrow depression of chronic sepsis, malignancy, or chemoradiotherapy. As the fluid balance is difficult to assess even with a central line to observe venous filling pressures, and a urinary catheter, daily weighing is extremely desirable.^20^

## MALNUTRITION

Anorexia, restricted oral intake, significant protein, electrolytes, and fluid loss from fistula effluent as a result of loss of unabsorbed small bowel secretions, may ultimately result in malnutrition.^2^ In high output fistula, there is a massive loss of ingested nutrients as fistula effluent an effectively causes short bowel syndrome with resulting intestinal failure. Lastly, patients often have increased energy demands because of ongoing sepsis and inflammation, countered by decreased demands due to immobility.^21^

## ENTERAL VERSUS PARENTERAL FEEDING

Nutritional support decreases or modifies the composition of the GI secretions and plays a primary therapeutic role. TPN or enteral nutrition is one among the corroborative cares to stop subsequent malnutrition and dyselectrolytemia.^21,22^

Gastrointestinal secretions decrease by 30-50 percent in patients getting TPN thus helping in fistula closure. There is ongoing debate about the effectiveness of either enteral or parenteral nutrition and if the periods of 'bowel rest' are advantageous in fistulas.^23^ The practice of total parenteral nutrition (TPN) is adopted exclusively for enterocutaneous fistula. The role of both is to provide nutritional support for avoiding malnutrition. Clear advantage for TPN within the fistula healing is not agreed upon currently, but it has its complications of central venous catheter sepsis, phlebothrombosis, and pneumothorax.

After elective gastrointestinal surgery, early enteral feeding has been shown to lower complication rates and decrease hospital stay as compared to Nil per Oral regimens.^24^ Early enteral feeding decreases the danger of anastomosis site leakage by improving anastomotic healing and increasing anastomotic strength compared to parenteral feeding, possibly because of trophic effects on the intestinal mucosa.^25^

Developed by Dr Etienne Levy in the 1970s, chyme reperfusion within which an extracorporeal circulation of the chyme between the gathering pouch and distal small intestine is completed. It amends intestinal failure, restores enterohepatic cycles, and stimulates L-cell enterocytes in proportion to the extra function that the downstream intestine can perform.^26^

Parenteral supplementation compensates for nutrients, water, and electrolyte losses. Chyme reperfusion artificially restores the continuity of the remaining intestine. The nutritional status, liver test abnormalities improve, and total halt or drastic reduction in IVS requirements also decreases the danger of central venous line complications. CR is also recommended by European Society for Clinical Nutrition and Metabolism (ESPEN) and the American Society for Parenteral and Enteral Nutrition (ASPEN) whenever possible.^26,27^

Uncontrolled sepsis remains the major factor contributing to mortality in patients with small intestinal fistulas. The aggressive management of continuing infections and careful observation for new septic foci are necessary for successful management. Malnutrition in the presence of uncontrolled sepsis cannot be treated without effective drainage of the septic source. It is important to forestall the severe local skin excoriation that develops around the site of an ECF. Fistulas that are controlled with a tube should cause minimal injury to fascia, subcutaneous tissue, and skin.

## EFFLUENT MANAGEMENT

The successful management of effluent can have a big impact on volume status, electrolyte balance, nutrition, and skin integrity.^28^ Adjunct medical management of ECF effluent has traditionally focused on two main areas: acid neutralization and volume reduction. The utilization of proton pump inhibitors can accomplish both goals and therefore the dose should be titrated until the effluent's pH is larger than 6 and the volume of output is less than 1L/day.^4^ Breaching the cycle of tissue inflammation, infection, and sepsis plays a key role within the approach of those complex patients.^5^

With negative pressure applied to the wound, the Vacuum-Assisted Closure (VAC) apparatus allows for excellent control of drainage, minimizes the scale of the abdominal wound, simplifies management by decreasing the frequency of dressing changes, and will promote healing of the fistula.^3^

## PHARMACOLOGIC SUPPORT

Somatostatin, a 14-amino acid peptide hormone, inhibits pancreatic exocrine secretions by decreasing the volume of pancreatic juice.^29^ Somatostatin agonists like Octreotide promotes earlier closure of fistula than TPN alone, even with malignant enterocutaneous disease, and is overall helpful in declining secretions in high-output fistulas to a convenient level.^30,31^

Proton pump inhibitors or histamine H2-receptor antagonists decrease gastric acid production, increase the transit time, and lower gastric secretions. These drugs help lessen fistula output, chiefly proximal fistulas or when there are high gastric secretions. Antiperistaltic agents like loperamide are useful in lowering intestinal transit times and decreasing fistula effluents.^32^ Refractory fistulas of Crohn's disease are successfully treated with short courses of cyclosporine and other immunosuppressive drugs.^33^

After stabilization is accomplished within 24-48 hours, the investigation usually takes place over the subsequent 7-10 days. Investigation implies an intensive evaluation of the digestive tube, definition of the anatomy of the fistula, and identification of any complicating features like abscess, stricture, or distal obstruction. Investigative studies should be designed to work out the presence and location of the fistula and to supply information regarding its cause.^34^

## INVESTIGATION

The investigation is the next phase of management. An intensive evaluation of the digestive tract, defining the anatomy of the fistula, and identifying any complications like stricture, abscess, or distal obstruction.

Various laboratory studies may be performed in the evaluation of an enterocutaneous fistula (ECF):

- Serum electrolytes, Complete blood count (CBC), Liver function tests.
- Serum transferrin - Low levels (< 200mg/dL) are a predictor of poor healing
- Serum C-reactive protein (CRP) - Levels could also be elevated

Fistulography is conventionally performed 7-10 days after the presentation of an ECF and helps to assess the length of the tract, the extent of the bowel-wall disruption, location of the fistula, and presence of the distal obstruction. Fistulograms have been the first choice in the assessment of ECFs as it provides a rapid and straight method of connecting a cutaneous opening with the digestive tract. In patients without sepsis, fistulograms could be the only imaging study needed.^35^

## COMPUTED TOMOGRAPHY

Computed tomography (CT) is useful in demonstrating any intra-abdominal abscess cavities. Such cavities can develop if an ECF has an indirect tract when it first drains into an abscess cavity and then drains to the outside cavity. Interloop abscesses might also be present, if an ECF is related to intra-abdominal sepsis.

Endoscopic evaluation, including colonoscopy, esophagogastroduodenoscopy, and ERCP, could also be helpful in certain specific clinical situations.^36^ For effective prevention and definitive treatment of enterocutaneous fistulas, knowing the causes and risk factors for fistula formation is the most. The issues linked with formation and preservation of entero-cutaneous fistulas (ECFs) are conventionally listed within the mnemonic contraction FRIENDS (F foreign body, R radiation, I infection or inflammatory bowel disease, E epithelialization, N neoplasm, D distal obstruction, and S short tract <2cm); and for the factors related to the development of Entero-Atmospheric Fistula are remotely defined and multiple factors are suggested like anastomotic disrupture, exposure of dehydrated and desiccated bowel to equipment and material used for temporary abdominal closure, severe wound infections, bowel ischemia, severe trauma, adhesions, bowel trauma during dressing changes, and negative pressure wound therapy.^36,37^

## DECISION

The organ of origin of the fistula is important in predicting spontaneous closure but was not associated with surgical closure or death. Water-soluble contrast material is run through the fistula, orally, and rectally to define the anatomy of the fistula prior to planned surgery. It helps in getting information about the anatomy of fistula, the proximal bowel length, and the quality of the remaining bowel.^38^

Timing of surgery: Septic foci should be effectively treated, and a decent clinical and nutritional condition are met before planning any definitive surgery. Patient should be mobile, feeling well, taking an interest in his/her surroundings, and be ready mentally to proceed with the restorative surgery. There should be an increase in albumin and haemoglobin levels and reduction in leukocyte and thrombocyte counts, C-reactive protein and erythrocyte sedimentation rate levels. The minimal period between the development of the fistula and the restorative surgical approach is around 6 weeks.^38,39^ Operating within 2 weeks or after 3 months has been shown to be related to lower fistula recurrence rate compared to if surgery is performed between 2 and 12 weeks. Early identification of a fistula is the most important significant commencement in management of those patients. Finally, where possible, enteral feeding should be provided and efforts should be made to start out early.^39,40^ Determining the optimal time for surgical intervention has not been well defined in the literature. Surgery may be postponed till the intra-abdominal and systemic circumstances of the patient are favourable to surgery. The timing of conclusive surgery must be customized according to patient features.^41^

## DEFINITIVE THERAPY

There is a higher chance of breakdown and recurrence of fistula when direct closure of fistula by suturing is completed. Primary end to end anastomosis after resection of the fistulous segment is the preferred surgical procedure of choice in most cases. Exteriorization of both the segments of the intestine is also required when there is extensive sepsis. Standard Brooke stoma should be constructed for proper fitting of appliances for efficient management of the effluent.

When a fistula develops as a complication of any deep pelvic surgeries, two or three staged approaches involving bypass surgery could also be required instead of simple resection anastomosis to avoid recurrence. A side-to-side anastomosis proximal and distal to the fistula is not enough, nor the unilateral exclusion of the involved segment. For effective defunctionalization of the fistula bilateral exclusion with isolation of both the proximal and distal portions of the involved intestine is mandatory.

In a staged procedure, the fistulous segment is left in place, then both the ends are externalised as mucous fistulas; and later the afferent and efferent bowel loops are anastomosed to revive intestinal continuity.^14^ Otherwise, if the efferent loop cannot be mobilized, the intestine proximal to a distal ileal fistula could also be divided and anastomosed to the transverse colon. And the fistulous part is again returned to the pelvis or externalised as a mucous fistula. Though not as efficient as complete exclusion but works well in the competent ileocecal valve. Later, the staged surgery is accomplished when the fistula segment is removed which is not always possible.

In conclusion, the surgical management of an ECF is technically challenging and the achievement rests on proper intraoperative decision and detailed preoperative optimization. Although nonoperative management may allow fistulas to heal spontaneously, the majority of those who fail within the first 4 weeks after development will require operative intervention.^40^

## VACUUM ASSISTED CLOSURE (VAC)

VAC has several added benefits in patients with ECF of which the major advantage is the ability to contain the effluent, protect the skin around the fistula, prevent further tissue breakdown and improve dermatitis.^41^

To put on the dressing, the encircling skin is first protected by a skin barrier, followed by the application of a transparent film. A foam filler is then cut and moulded to suit the contours of a wound bed and then taped up with a transparent film. A drainage tube is connected to the dressing through the gap of the transparent film which is connected to the vacuum. With this technique of management, nursing care and support is required for maintaining proper tissue viability.^42,43^

## ABDOMINAL CLOSURE DURING SURGERY

As per guidelines from International Endohernia Society (IEHS), for laparoscopic treatment of ventral and incisional abdominal wall hernias), mesh repair should be considered as the first choice for the repair of primary defects larger than 2cm or recurrent hernias of any size. If the ventral hernia is less than or equal to 2cm, ECF excision is done and abdomen closed. If the ventral hernia is > 2cm, the ECF excision is done and a selective repair of abdominal wall hernia or another planned ventral hernia repair is performed later to avoid infection caused by patch implantation.^44,45^

## FIBRIN SEALANT

In their study of 23 patients who underwent fibrin sealant fistula closure, Avalos-Gonzalez J, et al. demonstrated fistula closure at 12.5 days in the treatment group versus 32.5 days in the control group.^46^ Another study reported a series of 15 patients who underwent fibrin sealant measures, leading to an 86.6% healing rate at 16 days.^46^ Overall, fibrin sealant therapy could be used in selected cases with favourable configuration.

## ENDOSCOPIC CLIPS

Endoscopic clips are more suitable for acute fistulas compared to chronic ECF. Through-the-scope clips are used for repair of fresh injury with controlled sepsis and a small-sized fistula. This technique has lesser role in the cure of chronic ECF.^47^

## FISTULA PATCH

This technique attempts to seal the fistula from inside the bowel lumen; a soft, flexible gel lamellar is shaped in order to obtain a round shape and is then folded and pushed into the fistula. Once inside the bowel, it will unwrap, working like a patch. This dressing will remain in place until definitive fistula takedown surgery.^40^

## FISTULA PLUG

Fistula plugs have been more commonly used as an adjunct in the treatment of enteroatmospheric fistula (EAF). One case series of 6 patients using the Biodesign ECF plug described fistula closure in all patients followed by recurrence in two patients at 9 and 12 months.^1^

## 3D-PRINTED FISTULA STENT

Compared to fistula patch and fibrin glue, fistula stent goes together with the course and form of the ECF that minimises mechanical damage to the close by mucosa. It lowers fluid and electrolyte via effluent loss. The accrued effluent is reinfused from proximal bowel distally through the fistula via a feeding tube, fistuloclysis aid in decreasing the loss from fistula site. It has few drawbacks like fistula located too distally, fistula that are difficult to cannulate, or patient rejection. These downsides may be halted through the usage of 3D fistula stents because it has a patient-personalised layout and reinstated GI integrity.

Thermoplastic urethane (TPU) is a biocompatible material with supple mechanical properties and is used in medical devices such as catheters, pacemaker leads and vascular grafts. TPU fuses at 230-260 °C and is deposited at room temperature.^48^

A 3D-printed fistula stent is tailored under the guidance of 3D-reconstructed fistulography and is effective in decreasing effluent in ECF patients. This method is advocated though in addition research is wanted for gauging effective clinical outcome.^47^

## REHABILITATION

Development of an enterocutaneous fistula is usually an upsetting problem which results in protracted hospitalisation, pain, malaise, additional surgery, and key psychological indisposition. Psychological sequelae like depression, anxiety, guilt, and hospitalisation, may take many months to resolve. These difficulties should be accomplished by decent communication and understanding between the patient, the patient's family, and concerned health workers.^10^
